# Interfacial Electron Beam Lithography Converts an Insulating Organic Monolayer to a Patterned Single-Layer Conductor with Puzzling Charge Transport Performance

**DOI:** 10.1021/acsnano.4c02074

**Published:** 2024-07-09

**Authors:** Rivka Maoz, Peter Nelson, Bedanta Gogoi, Doron Burshtain, Santanu Talukder, Shuangyang Zou, Arup Sarkar, Jonathan Berson, Jacob Sagiv

**Affiliations:** Department of Molecular Chemistry and Materials Science, Weizmann Institute of Science, Rehovot 7610001, Israel

**Keywords:** organosilane monolayers, chemical e-beam lithography, single-layer electrical conduction, nanowires, Coulomb coupled transport, AFM, FTIR spectroscopy

## Abstract

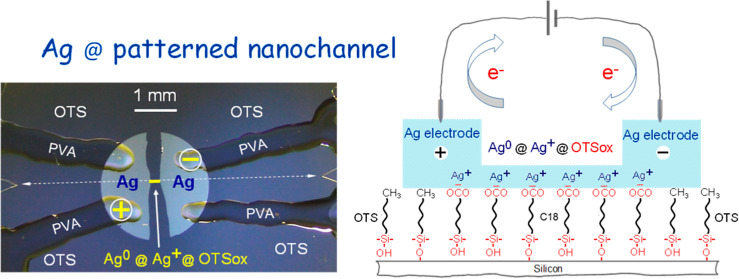

The direct generation of conducting paths within an insulating surface represents a conceptually unexplored approach to single-layer electrical conduction that opens vistas for exciting research and applications fundamentally different from those based on specific layered materials. Herein we report surface channels with single-layer –COOH functionality patterned on insulating *n*-octadecyltrichlorosilane monolayers on silicon that exhibit unusual ionic-electronic conduction when equipped with ion-releasing silver electrodes. The strong dependence of charge transport in such channels on their lateral dimensions (nanosize, macro-size), the type (p, n) and resistivity (doping level) of the underlying silicon substrate, the nature of the insulating spacer layer between the conducting channel and the silicon surface, and the postpatterning chemical manipulation of channel’s –COOH functionality allows designing channels with variable resistivities, ranging from that of a practical insulator to some unexpectedly low values. The unusually low resistivities displayed by channels with nanometric widths and micrometer-millimeter lengths are attributed primarily to enhanced electronic transport within ultrathin nanowire-like silver metal films formed along their conductive paths. Function–structure correlations derived from a comprehensive analysis of electrical, atomic force microscopy, and Fourier transform infrared spectral data suggest an unconventional mode of conduction in these channels, which has yet to be elucidated, apparently involving coupled ionic-electronic transport mediated and enhanced by interfacial electrical interactions with charge carriers located outside the conducting channel and separated from those carrying the measured current. These intriguing findings hint at effects akin to Coulomb pairing in the proposed mechanisms of excitonic superconductivity in interfacial nanosystems structurally related to the present metalized surface channels.

Interfacial electron beam lithography (IEBL)^1^ is a recently advanced mode of electron beam lithography applicable to chemical rather than topographic patterning.^1−12^ Exploiting interfacial chemical processes induced by electrons at the boundary between two solids,^13^ IEBL offers surface patterning capabilities particularly well suited for the local surface functionalization of *n*-alkylsilane monolayers while fully preserving their overall molecular organization and structural integrity. This was demonstrated in the nondestructive chemical patterning of highly ordered OTS/Si monolayers–monolayers self-assembled on smooth, native oxide-covered silicon wafer surfaces from *n*-octadecyltrichlorosilane precursor molecules [CH_3_–(CH_2_)_17_–SiCl_3_].^1,13^ In the IEBL process, the top –CH_3_ groups of OTS may be locally converted to –COOH without affecting the monolayer core made of densely packed –CH_2_– tail groups.^1^ The resulting patterns, denoted OTSox@OTS/Si, consist of oxidized monolayer regions with –COOH surface functionality (OTSox) seamlessly embedded within the inert –CH_3_ surface of the unmodified monolayer (OTS). Here, we report experimental results that bear evidence for a series of unusual aspects of electrical conduction associated with lateral charge transport on the –COOH surfaces of OTSox regions patterned by IEBL.

Monolayer regions with top –COOH functionality previously realized by local electro-oxidation of the top –CH_3_ groups of OTS/Si monolayers with conductive atomic force microscopy (AFM) probes^14−16^ (a form of oxidation scanning probe lithography, *o*-SPL^17,18^) or stamps^19,20^ as patterning tools were found to exhibit single-layer electrical conduction associated with fast lateral transport and exchange of protons and metal ions along the patterned –COOH surface paths.^21^ With the advancement of IEBL,^1^ it became immediately evident that the electron-beam mode of monolayer chemical patterning along with some additional experimental improvements allow fabrication of conductive OTSox features that outperform by far those previously produced by electro-oxidation (OTSeo) with conductive AFM probes. Attempts to identify factors responsible for the observed differences have soon evolved into an exploratory research effort that keeps generating unexpected results.

The electrical conduction of IEBL-patterned monolayer regions with –COOH surface functionality depends on many system parameters that may be deliberately varied and combined, part of which involving postpatterning chemical modification of the –COOH functionality installed in the patterning process. This offers various possible routes for realization of planned surface channels with variable electrical conduction. As shown in the following, using this approach, the room temperature resistivity of such channels equipped with silver electrodes could be modulated between values characteristic of a practical insulator to more than 3 orders of magnitude lower than that of silver - the most conducting metal. Much of these findings, in particular the observed dependence of channels’ electrical conduction on the material used as monolayer substrate and the unusually low resistances displayed by some of them may not be rationalized in terms of conduction mechanisms applicable to conventional electrical conductors. Therefore, we have embarked on an exploratory research effort aiming at a sufficiently rich body of experimental data that would eventually reveal relevant features of the conduction mechanism in this unconventional single-layer system. As it turns out, however, many of the experiments designed to elucidate a particular issue have in fact added unexpected findings to a growing body of intriguing experimental results.

Herein we report a series of experimental results obtained with conductive surface nanochannels and macrochannels fabricated by IEBL^1^ and the analogous macroscale chemical patterning of OTS monolayers via exposure through a mask to the radiation generated in a e-beam metal evaporator.^13^ For brevity, IEBL will be used in the following to refer to both the nano- and macroscale patterning processes. Within the context of this work, nanochannels are patterned OTSox@OTS lines with lengths between tens of micrometers to ca. 3 mm and widths of 10–50 nm, whereas the macrochannels are 16.5 mm or 22 mm long and 6.5 mm wide OTSox@OTS rectangles, both equipped with pairs of silver electrodes. The distance between the electrodes along an OTSox line or rectangle defines the length of the respective channel (Figure 1). Nanochannels’ widths are taken as the half-height AFM widths of the respective OTSox lines (Figure 2). Macrochannel setups in which an insulating spacer layer (ISL) separates the patterned monolayer from the silicon surface (denoted OTSox@OTS/ISL/Si) have also been investigated, their electrical conduction being compared with that of the basic OTSox@OTS/Si monolayer configuration.

**Figure 1:**
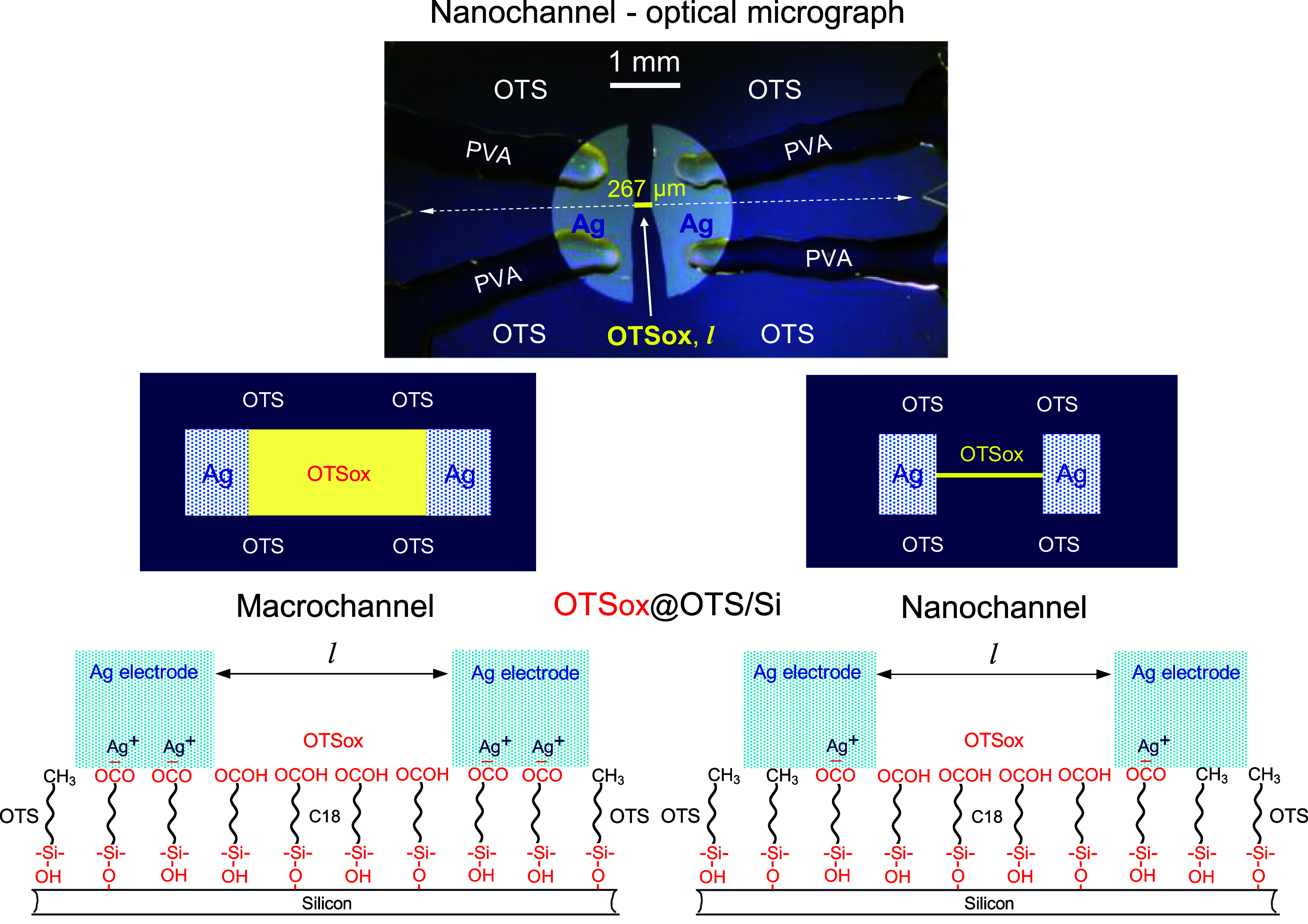
Optical micrograph of a OTSox@OTS/Si nanochannel specimen equipped with soft Ag/PVA pads (top) for nondamaging electrical contacts to the electrodes and schematic top views and molecular side views (not to scale) of a OTSox@OTS/Si macrochannel (left) and nanochannel (right) (see text). The 267 μm—long yellow line in the micrograph shows the position and length between the Ag electrodes of the OTSox line (i.e., nanochannel length, *l*) written with the electron beam along the dashed white line connecting the scratch markers at the left and right sides of the silicon substrate. It should be noted that the silver electrodes in the macrochannel are deposited exclusively on the patterned OTSox rectangle, whereas in the nanochannel the electrodes necessarily reside mostly on the OTS surface (−CH_3_) around the OTSox line. In the molecular schemes this is indicated by the conversion of –COOH to –COO^–^Ag^+^ in the OTSox areas covered by electrodes, which occurs spontaneously upon silver evaporation on the –COOH surface.^21^

**Figure 2:**
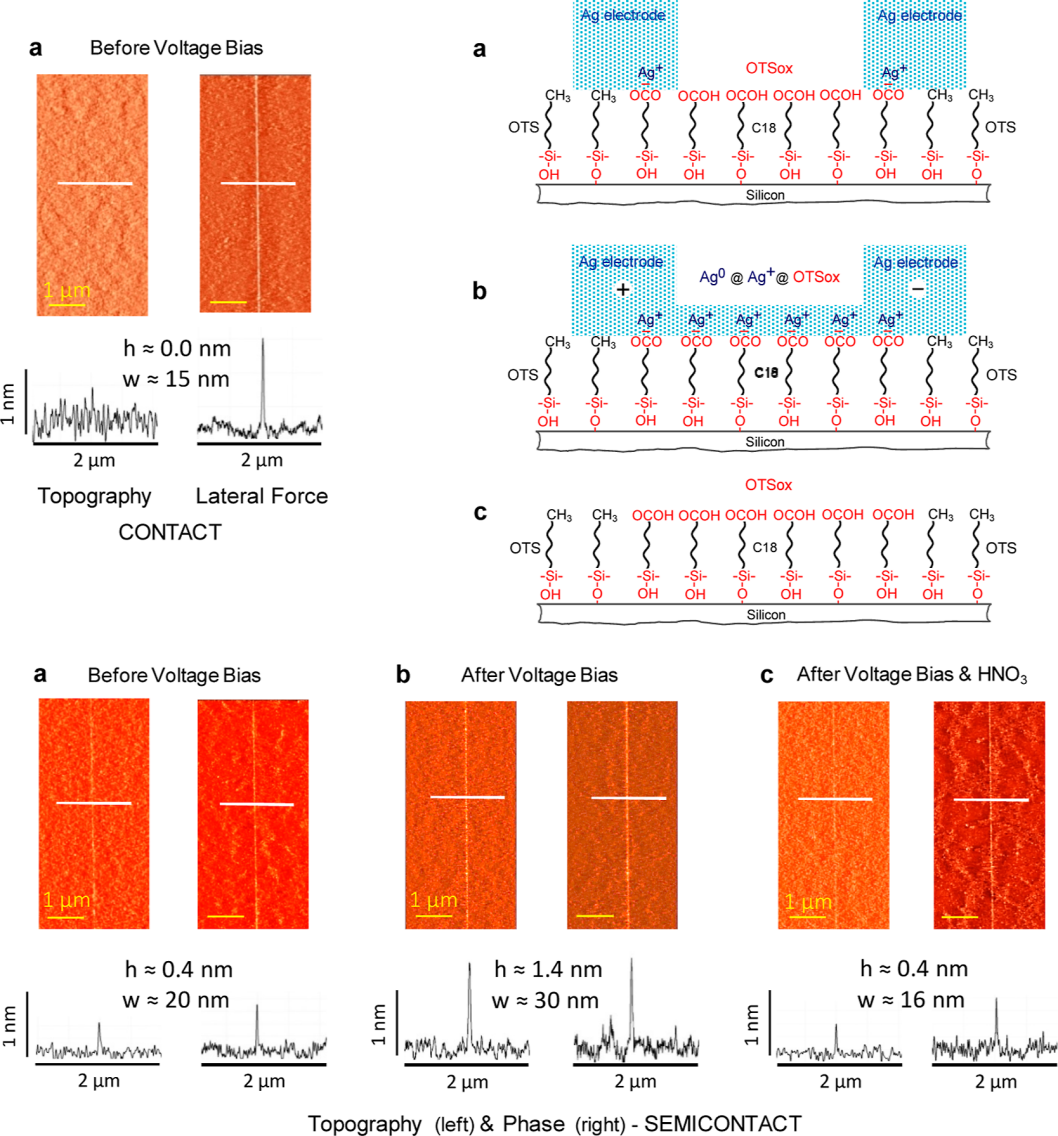
AFM contact (top) and semicontact (bottom) images of portions of a OTSox@OTS/Si nanochannel on the high-resistivity p-Si substrate (OTSox line written with an IEBL line width input of 0 nm) and corresponding schematic molecular side views of the nanochannel: (a) images of the as-patterned OTSox line, acquired before the application of a voltage bias. (Scheme a) depicts the –COOH top functions of OTSox; (b) images acquired after current flow upon the application of a dc voltage bias of 1 mV. (Scheme b) depicts the conversion of –COOH to –COO^–^Ag^+^ with the concomitant deposition of a thin silver metal film along the OTSox line (Ag^0^@Ag^+^@OTSox) upon the application of a voltage bias and current flow (see text); (c) images acquired after current flow upon the application of the voltage bias followed by treatment with nitric acid (HNO_3_). (Scheme c) depicts the regenerated –COOH surface functionality of the OTSox line, after removal of the silver electrodes and the silver metal deposited along it (scheme b) by dissolution in HNO_3_. The AFM cross-section profiles show channel heights (h) above the unmodified OTS surface and the respective half-height widths (*w*). Both contact (top) and semicontact mode (bottom) images are needed for a correct interpretation of the various observed AFM features^1^ (see text).

Combining the study of nanochannels with that of macrochannels bears particular relevance to the elucidation of structure–function relationships, by correlating Fourier transform infrared (FTIR) spectral information about chemical functionality and molecular structure collected from macrochannels with nanochannels AFM data that provide nanoscale structural information beyond the molecular composition and structure revealed by infrared spectroscopy. The joint application of these analytical tools is particularly well suited for a comprehensive nondestructive structural-chemical characterization of patterned molecular films over a wide range of length scales.^1,13,21^ Results presented here offer evidence for the dependence of channels’ electrical conduction on the type and conductivity of the silicon substrate, channel size, the presence and nature of an ISL between the conducting channel and the silicon surface, and the postpatterning chemical modification of channel’s –COOH functionality. An essential part of this report is devoted to control experiments designed to rule out possible artifacts and misinterpretations that might account for what appears as puzzling experimental results.

The overall experimental evidence presented here confirms our initial hypothesis as to the bias-induced transport of mobile cations (here H^+^ and Ag^+^) along the dense arrays of immobile carboxylate anions –COO^–^ resulting from the ionization of the –COOH top functions of OTSox.^21^ Ionic transport, however, represents only one contributing component to a considerably more complex mode of coupled ionic-electronic conduction dependent on continuous supply of mobile cations to the channel. The focus in the present work on silver as model electrodes that may release highly mobile Ag^+^ ions upon the application of a small voltage bias was prompted by the results of a previous series of experiments with structurally related channels fabricated by electrooxidation, in which by far the highest electrical conduction among several tested combinations of different electrodes (Ag, Ti, Al, Au, indium tin oxide) and mobile metal ions (Ag^+^, Na^+^, Ca^2+^, Al^3+^, Ti^3+^, In^3+^) was achieved with Ag^+^ ions supplied by pairs of silver electrodes.^21^

Considering trends identified in this study, the prospects of achieving ever-higher electrical conduction by proper engineering of the composition and structure of conductive surface entities produced by the present methodology appear most intriguing. We therefore believe the well-established factual evidence discussed here ought to be shared with the wide scientific community, even more so as it points to interfacial modes of electrical conduction in these unconventional single-layer conducting entities apparently related to phenomena such as coupled electron–hole transport and electron–hole exciton supercurrents involving spatially separated electrons and holes in adjacent conductive layers.^22,23^

## Results and Discussion

All OTSox@OTS lines (nanochannels) reported here were written using a thin poly(vinyl alcohol) (PVA) film as solid oxidant in the IEBL process.^1^ The OTSox@OTS rectangles (macrochannels) were fabricated by the analogous macroscale chemical patterning of OTS monolayers via exposure of the monolayer coated with a thin PVA film through a contact mask to the radiation emitted in a e-beam metal evaporator.^13^ Multimode AFM imaging^1^ and quantitative Brewster’s angle FTIR spectroscopy^21,24^ were routinely employed for the characterization of each patterned line and rectangle, which allowed optimization of the patterning process and assessment of its reproducibility.^1^ All electrical transport measurements summarized here were performed at the ambient temperature in a pure nitrogen environment (RH ≈ 2%) by recording current/resistance upon the application of a small dc voltage bias (typically 1.0 mV) to the pair of silver electrodes deposited on the patterned OTSox line or rectangle (Figure 1, Methods). Structural/chemical transformations resulting from the passage of current and postpatterning chemical operations (before and after the passage of current) were monitored by recording AFM images or FTIR spectra of the respective OTSox line or rectangle before and following each such operation.

### OTSox@OTS/Si Channels on p-Si and n-Si Substrates

A series of preliminary test studies with identically fabricated OTSox@OTS/Si channels on different silicon wafer substrates have revealed strong dependence of the measured electrical conduction on the type of silicon used as substrate. Lower channel resistance was found to correlate with lower silicon resistivity (higher doping level) on both n-type and p-type Si substrates (vide infra), however, depending on channel size and other parameters that affect electrical conduction (vide infra), channel resistances on p-Si were found to be ca. 25–300-fold lower than those of their counterparts on n-Si substrates with similar resistivities. These observations prompted us to focus efforts on a systematic investigation of channels on p-Si substrates before carrying out a related study with n-Si substrates. Most results presented here thus pertain to channels on p-Si substrates.

### OTSox@OTS/Si Nanochannels on p-Si Substrates with Different Resistivities

Examples of AFM contact and semicontact mode images of portions of OTSox@OTS/Si nanochannels on a high-resistivity (8–12 Ω cm) and a low-resistivity (1–5 × 10^–3^ Ω cm) p-Si wafer substrate recorded before and after current flow upon the application of a voltage bias of 1 mV, are given in Figures 2 and S1–S3. All nanochannels reported here were fabricated under IEBL conditions (Methods) that were previously found to afford quantitative (or close to quantitative) nondestructive conversion of –CH_3_ (OTS) to –COOH (OTSox).^1^ This is confirmed by the following characteristic features in the AFM images of the as-patterned OTSox lines^1^ (images “Before Voltage Bias” in Figures 2a, S1 and S2 top, and Figure S3 top): (i) high friction contrast between the patterned OTSox line and the unmodified OTS surface (lateral force images, contact mode) along with absence of OTSox heights in excess of ca. 0.4 nm above the surrounding OTS surface (real contact mode heights being given by the average of topography trace and retrace scans^1,20^); (ii) inverted OTSox vs OTS contrast in simultaneously acquired contact topography and lateral force images, both of which also changing sign between trace and retrace scans.^1^

According to topography images recorded before and after the application of a voltage bias (Figures 2a,b, S2, and S3), the AFM heights of all nanochannels increase by ca. 1 nm following current flow, thus indicating deposition of elemental silver (Ag^0^) by the electrochemical reduction of Ag^+^ ions moving on the dense arrays of immobile –COO^–^ anions resulting from the ionization of the –COOH surface functions of OTSox upon the application of a voltage bias^21^ (Figure S4). That the deposited material is elemental silver was confirmed by its dissolution in aqueous HNO_3_ (compare nanochannel heights in the topography images in Figure 2a–c) as well as by the treatment with a silver enhancer solution, which results in further deposition of silver metal under the catalytic action of preexisting Ag^0^ grains.^25,26^ Within the accuracy of the AFM measurements (vide infra), the same ca. 1 nm height increase was observed after seconds to many hours of current flow, which points to a rapid self-limited process of silver metal deposition along the nanochannels.^21^

To minimize possible mechanical damage/removal of the deposited silver by the AFM tip, only semicontact (tapping) AFM images were collected following current flow (Figures 2b,c, S2 and S3). For consistency, semicontact images were also acquired before current flow, as semicontact heights and widths tend to be somewhat larger than those in the respective contact mode images (e.g., Figure 2a bottom vs Figure 2a top). Along with the height increase, significant nanochannel widening (beyond the ca. ± 5 nm variations of the measured AFM widths—vide infra), has been observed following current flow, the widening being relatively more pronounced the narrower the as-patterned OTSox line (compare the AFM widths in images “before voltage bias” and “after voltage bias” in Figures 2, S2 and S3). This may point, along with the reversal of nanochannels’ heights and widths following the HNO_3_ treatment to their initial values before current flow (the *h* and *w* values in Figure 2c compared to those in Figure 2b,a), to mushroom-like deposition of silver beyond the edges of the patterned –COOH paths that do not change in the process.

IEBL allows routine fabrication of conductive nanochannels exceeding millimeter lengths, whereas AFM images may capture only very small portions of their full lengths. AFM provides essential structural information about the patterning process and subsequent transformations resulting from various postpatterning operations (vide infra), however, it is short of telling much about patterned features significantly larger than the dimensions covered by AFM scanners, which, depending on the resolution desired, may vary between several to ca. 100 μm. Such information has been obtained in the present study from electrical transport measurements, by checking the dependence of electrical conduction on channel dimensions.

**Figure 3:**
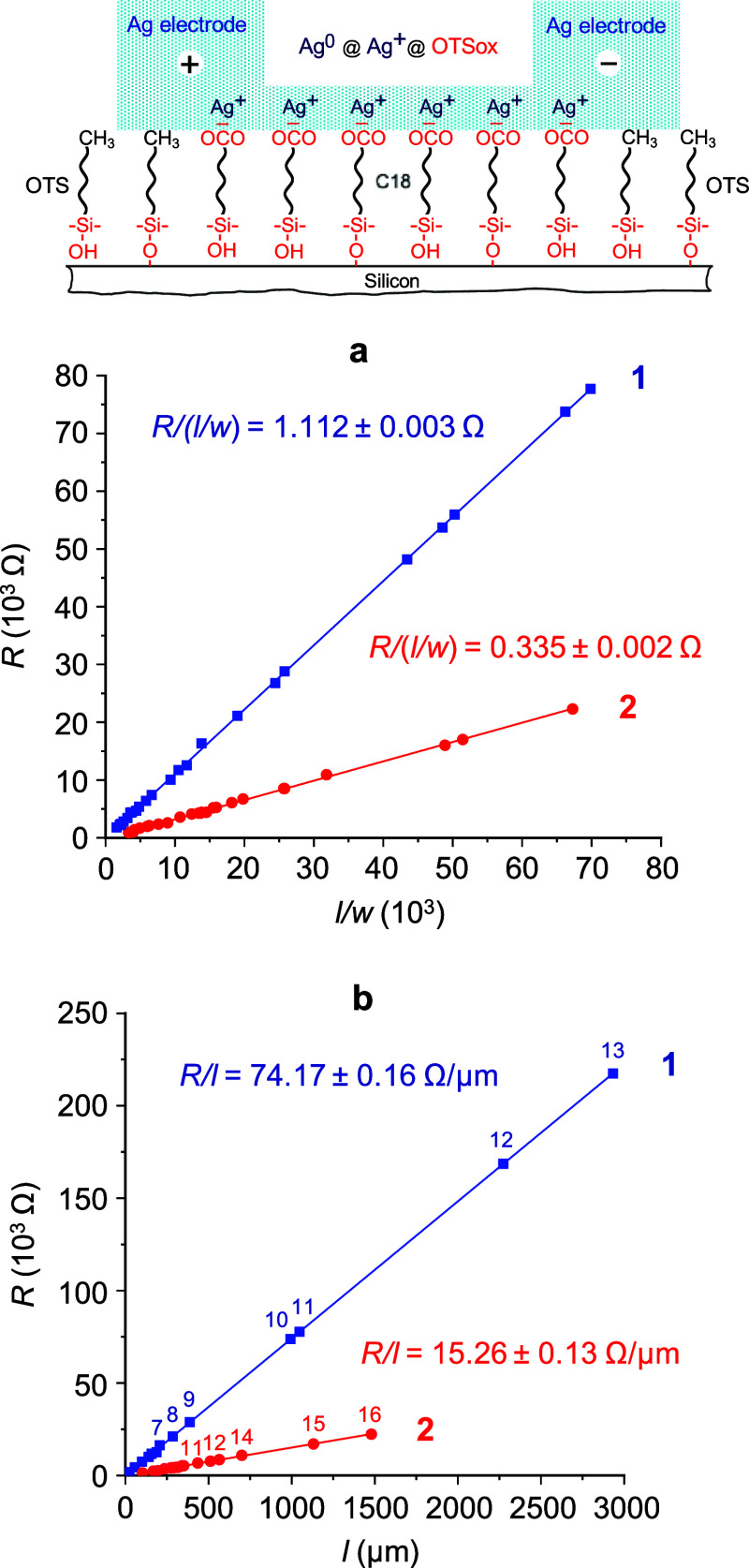
Plots of the electrical resistance (*R*) vs nanochannel length/width (*l*/*w*) ratio (a) and electrical resistance vs nanochannel length (b) of OTSox@OTS/Si nanochannels on the high-resistivity (1) and low-resistivity (2) p-silicon substrates (at an applied dc voltage bias of 1 mV). The schematic molecular side view of a nanochannel following the application of a voltage bias and current flow (top) depicts, as in Figure 2b, the composition and structure of the nanochannels during the measurement of their electrical resistance (see text). The lengths and as-patterned widths (before current flow) of the nanochannels in plot a vary between 24 μm ≤ *l* ≤ 1048 μm and ca. 15 nm ≤ *w* ≤ ca. 45 nm (1600 ≤ *l*/*w* ≤ 69,867) on the high-resistivity silicon (1) and 73 μm ≤ *l* ≤ 1480 μm and ca. 20 nm ≤ *w* ≤ ca. 40 nm (3338 ≤ *l*/*w* ≤ 67,273) on the low-resistivity silicon (2). The as-patterned widths of the nanochannels in plot b are ca. 15 nm (1) and ca. 22 nm (2) (see text).

The linearity of nanochannel resistance (*R*) vs nanochannel length/width (*l*/*w*) plots (Figure 3a) offers evidence for the confinement of charge transport to continuous OTSox paths with uniform effective widths over their full lengths between the electrodes to which the voltage bias is applied. Accordingly, each line slope, *R*/(*l*/*w*) = *r*_s_, represents the common sheet resistance (2D resistivity) of the nanochannels in the respective plot.^21^ A linear dependence of *R* on *l* (Figure 3b) was further found for a majority points in each of the *R* vs *l*/*w* plots in Figure 3a. As *R*/*l* = *r*_s_/*w*, nanochannels obeying both the linear dependence of *R* on *l*/*w* (Figure 3a) and *R* on *l* (Figure 3b) must have the same effective conduction width. Indeed, all nanochannels in each of the plots in Figure 3b have the same average AFM width; *w* = ca. 15 nm in plot **1** and ca. *w* = 22 nm in plot **2**. As it turned out, these are the average AFM widths of most OTSox lines written in the fixed-beam-moving-stage (FBMS) mode of the e-beam writer with a line width input of 0 nm, namely the narrowest OTSox lines patterned with the given electron dose on each of the respective silicon substrates (Methods).

The different points in the plots in Figure 3 represent both nanochannels derived from different OTSox lines and nanochannels obtained by redeposition of the electrodes at different *l* distances from one another (Figure 1) along the same OTSox line. The latter (Figure 3b, data summarized in Table 1) offer further compelling evidence for both the reproducibility of the IEBL patterning and the uniformity of the effective conduction widths of IEBL-patterned OTSox lines up to nanochannel lengths of several millimeters. Particularly noteworthy are the two extreme points (1 and 13) in plot **1** of Figure 3b, representing nanochannels with *l* = 24 μm and *l* = 2933 μm derived from same OTSox line, for which identical *R*/*l* values equal to the slope of the plot were obtained despite the huge difference in their lengths (Table 1). The preservation of the conduction of a given OTSox line upon repeated removal and redeposition of electrodes on it (which involves dissolution in HNO_3_ of both the electrodes and the silver metal deposited along the OTSox path between the electrodes, followed by thermal evaporation of new electrodes) further demonstrates the outstanding robustness of the conductive surface paths fabricated by the present process. This offers opportunities for system manipulation which may not be realized with conventional nanowire systems.

**Table 1:** Data Points in the *R* vs *l***Plots** in Figure 3b Representing Nanochannels with Different Lengths Obtained by Redeposition of the Electrodes at Different Distances (*l*) from One Another along the Same OTSox Line

OTSox line (**plot**)	plot point	*R* (Ω)	*l* (μm)	*R/l* (Ω/μm)
1 (**1**)	1	1780	24	74.17
	13	217,400	2933	74.12
2 (**1**)	2	4400	57	77.19
	8	21,110	285	74.07
	11	77,700	1048	74.14
3 (**1**)	3	7400	100	74.00
	12	168,600	2273	74.17
4 (**1**)	5	11,710	157.9	74.16
	9	28,820	387	74.47
5 (**1**)	7	16,320	207	78.84
	10	73,740	993	74.26
1 (**2**)	2	2354	168.3	14.00
	12	7721	512	15.08
2 (**2**)	11	6700	436	15.37
	16	22,290	1480	15.06

Whereas ionic transport is a prerequisite for electrochemical deposition of elemental silver along the nanochannels upon the application of a lateral voltage bias (Figure S4b,c, **Control Experiments**),^21^ it alone may not account for the sheet resistance values (*r*_s_) derived from the plots in Figure 3a, which are more than 3 orders of magnitude lower than what would be possible exclusively via ionic conduction in a single ionic layer.^21^ Therefore, we ascribe the observed electrical conduction mainly to transport within the nanowire-like metal paths produced by the rapid electrochemical deposition of elemental silver along the patterned OTSox lines (Ag^0^@Ag^+^@OTSox in Figures 2b, 3 top, and Figure S4c). These ca. 1 nm-thick belt-shaped silver deposits (Figures 2b, S2 and S3) have lengths and widths defined by the *l* and *w* dimensions of the respective nanochannels. Subject to these considerations, the linearity of *R* vs *l*/*w* and *R* vs *l* plots (Figure 3) implies that all nanowire-like silver deposits produced in this process must have the same effective conduction thickness (*h*), which, according to the AFM images, is of the order of 1 nm. By taking *wh* as a reasonable estimate of the conduction cross section of each of these metalized OTSox lines, we obtain *R* = *rl*/*wh*, where *r = r*_s_*h* is a reasonable estimate of their common resistivity (3D) in each of the plots in Figure 3a. With *r*_s_ = 1.112 Ω and *r*_s_ = 0.335 Ω (Figure 3a, plots **1** and **2**, respectively), and *h* ≈ 1.0 × 10^–7^ cm, the corresponding resistivities of these nanowire-like silver entities on the high- and low-resistivity silicon substrates are *r* ≈ 1.112 × 10^–7^ Ω cm (plot **1**) and *r* ≈ 0.335 × 10^–7^ Ω cm (plot **2**), respectively. These figures are obtained with the *w* values used in the plots in Figure 3a, namely the average AFM half-height widths of the respective OTSox lines measured before the application of the voltage bias. By taking into account their apparent widening following current flow, i.e., assuming effective nanochannel conduction widths up to twice larger than the respective as-patterned OTSox widths (Figures 2, S2 and S3), the corresponding resistivities would increase up to *r* ≈ 2.224 × 10^–7^ Ω cm and *r* ≈ 0.670 × 10^–7^ Ω cm. Compared to the room temperature resistivity of bulk silver metal, *r*_Ag_ ≈ 1.600 × 10^–6^ Ω cm,^27^ these nanowire-like silver resistivities are thus 14.4–7.2 and 47.8–23.9-fold lower, depending on the assumed variations of their effective widths and the type of silicon used as substrate. This is an unexpected result. It shows that (i) nanowire-like silver entities produced by the present process exhibit considerably enhanced conduction compared to that of bulk silver metal; (ii) the conduction enhancement depends on the type of silicon used as monolayer substrate, being ca. threefold higher in nanochannels on the low-resistivity compared to those on the high-resistivity p-silicon.

One should note that in contrast with the negligible experimental uncertainty in the measurement of nanochannels’ lengths from the respective optical micrographs (e.g., Figure 1), the relative uncertainties of their *w* and *h* dimensions determined by AFM may be very large. Cross section profiles taken with different tips and at different positions along a OTSox line may give up to ±5 nm variations in its measured half-height width, which translate into large possible variations of the respective *l*/*w* ratios. Such variations become larger the longer and narrower the nanochannel. For example, in the case of a nanochannel with *l* = 1.0 mm and *w* = 15 ± 5 nm, the *l*/*w* = 66.67 × 10^3^ ratio obtained with the average nanochannel width of 15 nm varies between *l*/*w*_max_ = 50.00 × 10^3^ and *l*/*w*_min_ = 100.00 × 10^3^; i.e., an uncertainty range of up to 75% of the average *l*/*w* value. Likewise, the ca. 1 nm AFM thickness of the silver deposited along each nanochannel is also an estimated average value with an experimental uncertainty of the order of ±0.4 nm. In the light of this analysis, the very good linearity of the plots in Figure 3, implying uniform *w* and *h* dimensions, is a striking result. Moreover, judging from the AFM images, the grainy structure of the silver layer deposited along the OTSox lines upon the application of a voltage bias was expected to result in a rather poor electrical conduction,^28,29^ just contrary to the unusually low nanochannel resistivities derived from the plots in Figure 3. This implies effective conduction paths with rather uniform cross sections, which necessarily must be smaller than those given by the average *w* and *h* dimensions derived from the AFM images. The AFM dimensions presumably include variable local surpluses of deposited silver that do not contribute to the measured electrical conduction, the effective *w* and *h* dimensions of the conduction paths being thus closer to the respective minimal rather than average AFM values. Accordingly, with *w* ≈ 10 nm and *w* ≈ 17 nm for nanochannels on the high- and low-resistivity silicon substrates, and *h* ≈ 0.5 nm, the corresponding resistivities, *r* ≈ 0.371 × 10^–7^ Ω cm and *r* ≈ 0.129 × 10^–7^ Ω cm, would be ca. 43-fold and ca. 124-fold lower than that of bulk silver metal!

The sheet resistance of present OTSox@OTS/Si nanochannels on the high-resistivity silicon (1.112 Ω, Figure 3a) is a factor of 173 lower than that previously reported for OTSeo@OTS/Si nanochannels fabricated on same silicon substrate by the electro-oxidation nanopatterning of OTS/Si monolayers with conductive AFM probes (192 Ω).^21^ In the electro-oxidation nanopatterning, the writing of continuous, defect-free OTSeo lines is hampered by the difficulty of maintaining a reproducible tip–surface water meniscus^30,31^ during pattern writing. Patterning defects caused by occasional breaking or thinning of the water meniscus, resulting in incomplete local conversion of –CH_3_ to –COOH, would necessarily impair the conduction of the patterned OTSeo lines. This becomes particularly critical the longer and narrower the nanochannel. The considerably higher conduction displayed by the present much longer and narrower nanochannels is thus attributed to the IEBL capability of writing extremely long defect-free OTSox lines, combined with the use of the nondestructive PVA peeling procedure^1^ for the cleaning of the patterned –COOH paths and their protection during the deposition of the silver electrodes (Methods).

### OTSox@OTS/Si and OTSox@OTS/ISL/Si Macrochannels on the High-Resistivity p-Si Substrate

**Figure 4:**
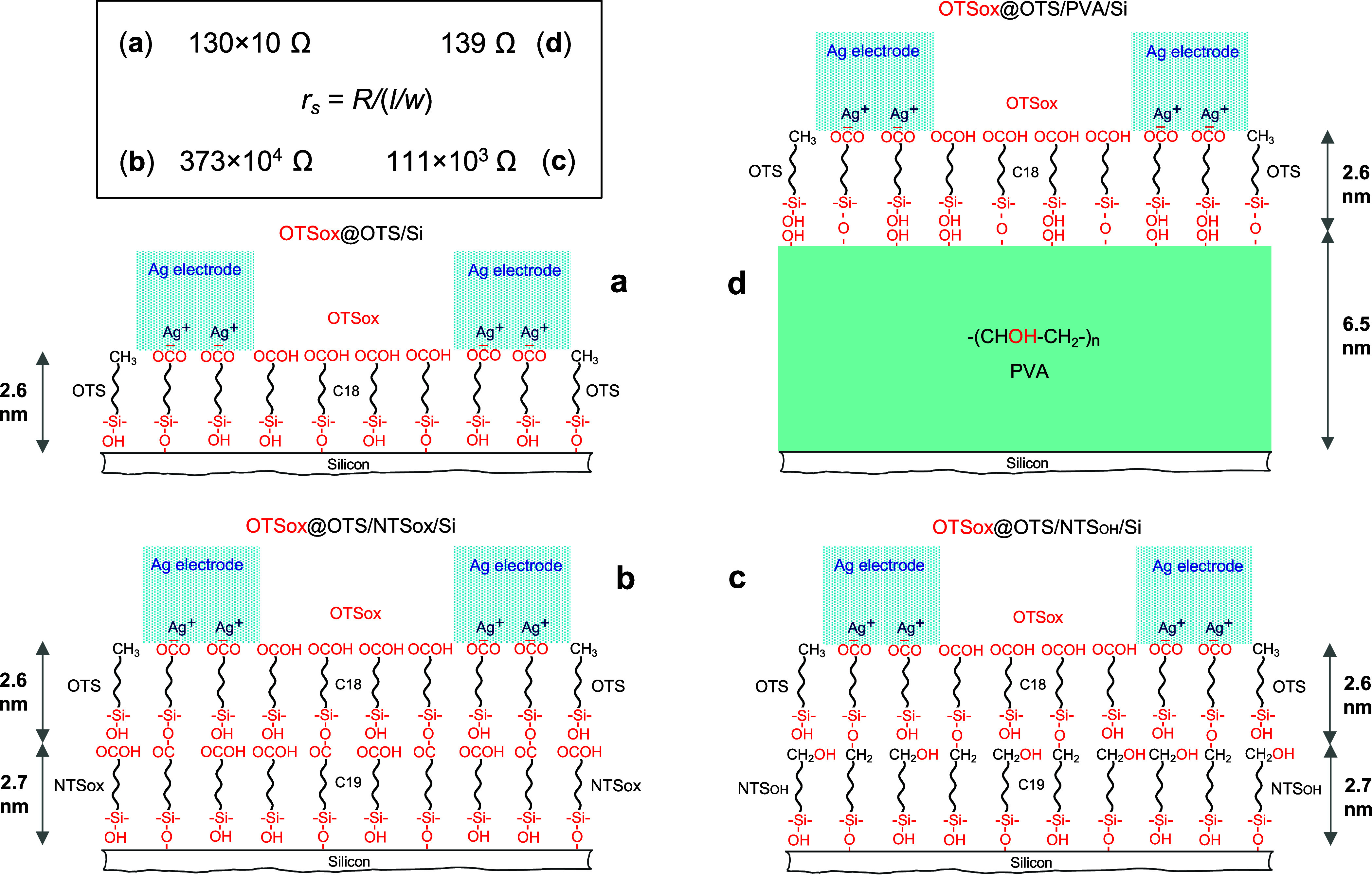
Schematic molecular side-views of macrochannel setups with different ISLs between the channel monolayer (OTSox@OTS) and the silicon substrate: (a) ISL-free monolayer setup; (b) bilayer setup with NTSox/Si monolayer (−COOH top functionality) as ISL; (c) bilayer setup with NTS_OH_/Si monolayer (−CH_2_OH top functionality) as ISL; (d) bilayer setup with a thin PVA/Si film (−CHOH– top functionality) as ISL. Note the partial covalent bonding (depicted schematically) between the OTSox@OTS monolayer and each of the underlying ISLs, as well as between the OTSox@OTS, NTSox, and NTS_OH_ monolayers and the native oxide layer on the silicon substrate. The sheet resistance (*r*_s_) values obtained with each of the respective setups are displayed in the top-left panel (see text).

**Figure 5:**
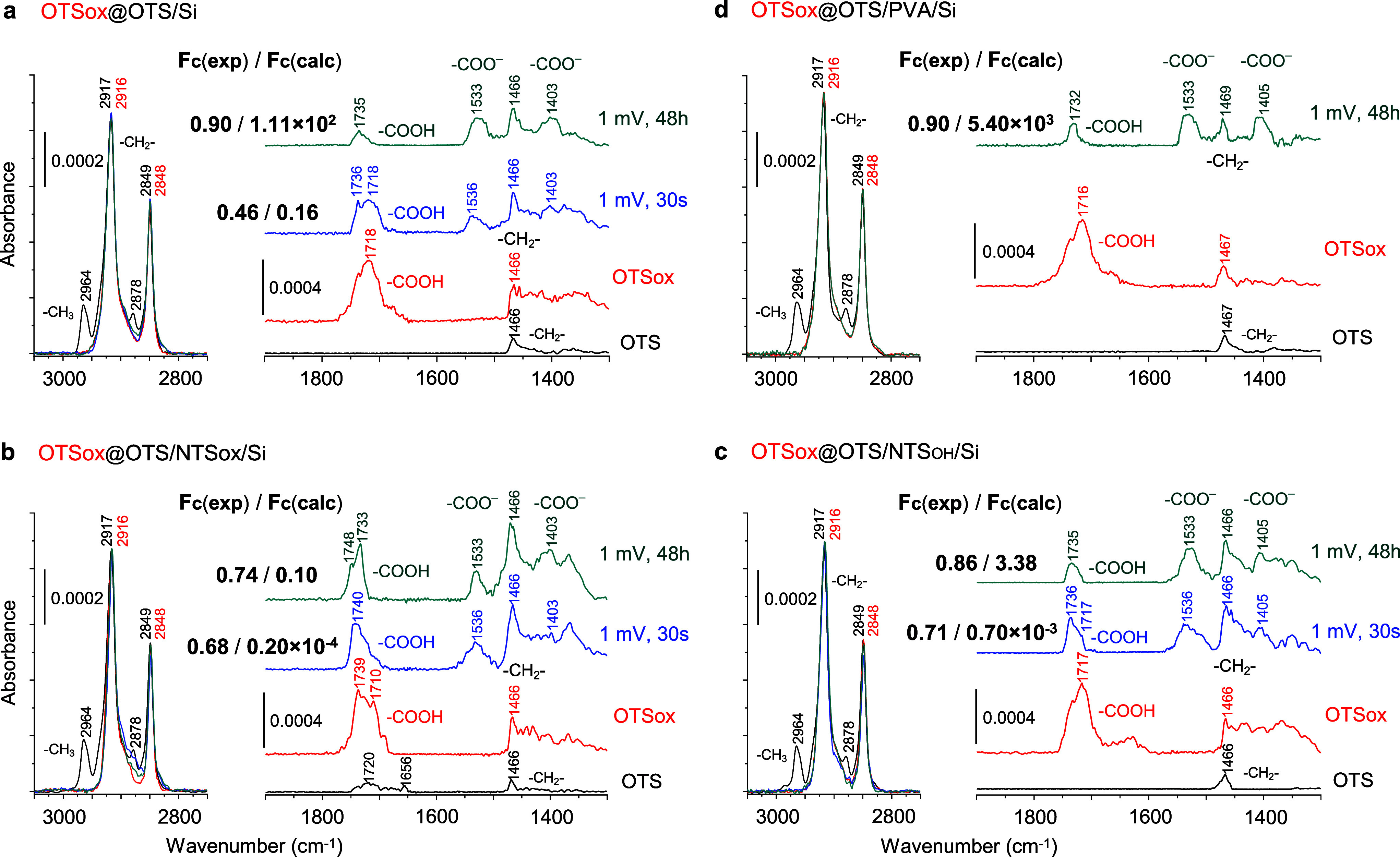
Quantitative Brewster’s angle FTIR spectra of the OTSox@OTS monolayer in each of the macrochannel setups displayed in Figure 4. OTS: OTS monolayer before patterning; OTSox: patterned OTSox channel region before current flow; 1 mV, 30 s: OTSox channel region after current flow for 30 s at a dc bias voltage of 1 mV; 1 mV, 48 h: OTSox channel region after current flow for 48 h at a dc bias voltage of 1 mV. All curves are difference spectra representing the net spectral contributions of OTS or OTSox before and following current flow, after mathematical subtraction of the contributions of the bare silicon substrate and the respective ISLs to the measured raw spectra. Fc(exp) is the fraction of channel area with –COOH groups converted to –COO^–^Ag^+^ upon current flow as determined experimentally from the FTIR spectral data; Fc(calc) is the corresponding maximal value calculated from the respective measured current and time of current flow (see text).

The finding that OTSox@OTS/Si channels on different silicon substrates exhibit different electrical conduction led us to check the effect of the distance from the silicon substrate on channel conduction. It was expected that the conduction enhancement observed with p-Si substrates would diminish the larger the distance of the channel from the substrate. To this end, we have carried out a comparative study of four macrochannel configurations: the basic OTSox@OTS/Si monolayer setup and OTSox@OTS/ISL/Si setups with three different ISLs between the silicon substrate and the OTSox@OTS monolayer (Figures 4, S5). Using macrochannels rather than nanochannels and by routinely collecting quantitative FTIR spectra^13,21,24^ from each setup before and after each stage in its fabrication as well as after the passage of current (Figure 5) allowed the measured electrical conduction to be unequivocally associated with the actual composition and structure of the respective setup. Subject to the need of an IR-transparent substrate, only high-resistivity p-silicon substrates were used in this study.

Two of the investigated ISLs, NTSox (Figure 4b) and NTS_OH_ (Figure 4c), are self-assembled *n*-alkyl silane monolayers (like OTS) with practically equal thickness defined by their molecular structure but different top functionality (−COOH and –CH_2_OH, respectively),^32^ whereas the third one, PVA, is a thin polymer film whose repeating unit includes the −CHOH- alcohol moiety (Figure 4d). According to X-ray reflectivity, X-ray photoelectron spectroscopy, and FTIR data, the NTSox and NTS_OH_ monolayers are 2.7 nm-thick, the interlayer bonding in the precursor OTS/NTS_OH_ bilayer being partially covalent and in the precursor OTS/NTSox bilayer exclusively via hydrogen bonds.^32^ As shown in the following, partial interlayer covalent bonds are also generated in the OTSox/NTSox bilayer upon the IEBL conversion of OTS to OTSox. Thus, we may reasonably assume the OTSox-ISL interlayer bonding is partially covalent in all three OTSox@OTS/ISL/Si setups (as schematically depicted in Figures 4 and S5). The 6.5 ± 0.5 nm thickness of the PVA film was assessed from the intensities of its characteristic infrared bands at 2943–2907 and 1430 cm^–1^ (Figure S6).^1,13^ All macrochannels in Figure 4 have the same dimensions (22 mm × 6.5 mm).

The sheet resistance (*r*_s_) values listed in Figure 4 show that the presence of an ISL between the silicon substrate and the conducting channel may indeed affect dramatically channel’s electrical conduction, however, not as expected. With NTSox as spacer layer, the sheet resistance of the conducting channel (373 × 10^4^ Ω, Figure 4b) becomes more than 3 orders of magnitude higher than that in the basic OTSox@OTS/Si channel configuration (130 × 10 Ω, Figure 4a), whereas with the equally thick NTS_OH_ spacer layer it is less than 2 orders of magnitude higher (111 × 10^3^ Ω, Figure 4c), and with the much thicker PVA spacer layer it actually drops to a value almost an order of magnitude lower (139 Ω, Figure 4d). It follows that the electrical conduction of the setup with the PVA spacer layer (Figure 4d) exceeds that with the NTSox spacer (Figure 4b) by more than 4 orders of magnitude! Thus, depending on composition and structure, an ISL may enhance rather than suppress channel conduction. Carboxylic acid functions in the spacer layer appear to contribute to the suppression of electrical conduction (Figure 4b), whereas alcohol hydroxyls have an opposite effect (Figure 4c,d). At present, we may merely speculate that different charge carriers generated during the IEBL process in ISLs with different compositions/structures might contribute to these differences. It is further evident that the ISL effect does not override that exerted by the underlying silicon substrate even in the case of the relatively thick PVA spacer layer. This is demonstrated by the 438 × 10^2^ Ω sheet resistance of an identical OTSox@OTS/PVA/Si macrochannel setup on a n-Si substrate with similar resistivity, which is more than 300-fold higher than that on the p-Si substrate (139 Ω, Figure 4d).

In this evaluation of the ISL effect, we have tacitly assumed that (i) the OTSox paths resulting from the IEBL patterning of OTS in the different examined channel setups are practically identical; (ii) the IEBL process converting OTS to OTSox does not affect significantly the structure of each of the underlying spacer layers. As demonstrated by quantitative FTIR spectral data collected from the different channel setups (Figure 5), these conditions are indeed largely fulfilled. The disappearance of the –CH_3_ stretch modes of OTS at 2964 and 2878 cm^–1^ in all OTSox curves along with the appearance of characteristic C=O stretch modes between 1800 and 1600 cm^–1^ (−COOH monomers around 1731–1736 cm^–1^, laterally hydrogen-bonded dimers around 1715–1718 cm^–1^, and oligomers at lower wavenumbers in the band tail extending below ∼1680 cm^–1^)^21^ bear evidence for the virtually quantitative conversion of –CH_3_ to –COOH in all channel setups. That both the conversion of OTS to OTSox and the passage of current through the OTSox paths do not affect the highly ordered structure of the respective monolayers is demonstrated by the virtual invariance (in all spectral curves in Figure 5) of the methylene H–C–H stretch bands at 2917–2916 and 2849–2848 cm^–1^, which are characteristic of densely packed alkyl tails in their extended all-*trans* conformation.^21,24,32^ The small but systematic shift of the 2917 and 2849 cm^–1^ band peaks of OTS to, respectively, 2916 and 2848 cm^–1^ in OTSox along with the slightly narrower widths of these bands in OTSox compared to OTS point to an even slight improvement of the organization and packing of the alkyl tails upon the conversion of OTS to OTSox.

One should further note that all OTS and OTSox spectral curves (before and following current flow) displayed in Figure 5 are difference spectra obtained by mathematical subtraction of the contributions of the silicon substrate and the respective ISLs to the measured raw spectra. This assumes invariance of the ISL upon both the assembly of OTS on top of it and then upon the conversion of OTS to OTSox and the passage of current through the channel. Post factum, this is largely confirmed by the similarity of the different OTS and OTSox curves in Figure 5, except for the carboxylic acid spectral region between 1800 and 1600 cm^–1^ in Figure 5b. Here the difference spectra representing OTS and OTSox necessarily reflect eventual changes in the carboxylic acid of the NTSox spacer layer as well. Thus, the weak features visible between 1750 and 1650 cm^–1^ in the OTS curve in Figure 5b actually arise from the enhanced –COOH features of NTSox due to the interaction with the silanol groups of OTS in the OTS/NTSox bilayer. Likewise, the partial formation of OTSox-NTSox interlayer covalent bonds accompanying the IEBL conversion of OTS to OTSox shows up in the OTSox curve in Figure 5b as the prominent 1739 cm^–1^ peak assigned to the C=O stretch mode in the –CO–O–Si- moiety.^33^ Accordingly, the corresponding loss of –COOH groups of NTSox upon the formation of such interlayer covalent bonds manifests itself in the OTSox curve as an apparent weakening of its –COOH features around 1717 cm^–1^ and the disappearance of those giving rise to the –COOH band tail below ∼1680 cm^–1^. Thus, the different –COOH spectral features in Figure 5b compared to those in Figure 5a,c,d reflect these changes in the mode of OTSox-NTSox interlayer bonding compared with OTS-NTSox rather than significant differences between the OTSox path in this channel setup and those in the other setups. The thickness of the NTSox spacer layer is not significantly affected by these changes in the mode of interlayer bonding.^32^

The gradual disappearance following the application of a voltage bias of the –COOH features between 1800 and 1600 cm^–1^ in all OTSox curves (Figure 5) and concomitant growth of silver carboxylate bands around 1533–1536 and 1400 cm^–1^ (the –COO^–^ antisymmetric and symmetric stretch modes, respectively) bear evidence for the bias-induced replacement of carboxylic acid protons by Ag^+^ ions supplied by the silver electrodes.^21^ As the –COOH conversion to –COO^–^Ag^+^ does not occur in the absence of a voltage bias, FTIR spectra recorded after the voltage bias is turned OFF represent “frozen” states of the system attained upon current flow for the indicated lengths of time. The ion exchange process revealed in this manner has been reasonably associated with ionic current involving the bias-driven lateral transport of mobile H^+^ and Ag^+^ cations on the lattice of immobile –COO^–^ anionic sites generated along the OTSox path upon the ionization of its top –COOH and –COO^–^Ag^+^ functions.^21^ Support to this ion transport scenario comes from control experiments that confirm the reversible loss and regain of channel’s electrical conduction following chemical reduction of the ionizable –COOH functions to –CH_2_OH and their back oxidation to –COOH (vide infra), as well as the loss of channel conduction upon the use of gold electrodes instead of silver or other ion-releasing metal electrodes.^21^ As shown in the following, however, our attempts to correlate the observed exchange of ions with the amount of electrical charge transported through the channel have led to another series of rather surprising findings.

Knowing the macrochannel area and the surface concentration of –COOH groups (5.0 × 10^14^/cm^2^; given by the 0.2 nm^2^ surface area of the OTSox molecule in the monolayer^32^), and assuming the measured currents to be entirely ionic, one may calculate the maximal fraction of channel area with –COOH groups converted to –COO^–^Ag^+^ following current flow for a given time, Fc(calc), by further assuming that as long as not all carboxylic acid protons are replaced by silver ions, each Ag^+^ ion entering the channel displaces one H^+^ ion that must exit the channel in order to maintain electroneutrality.^21^ The actual fraction of channel area with –COOH groups converted to –COO^–^Ag^+^, Fc(exp), is then estimated from the drop in the integrated intensities of the initial –COOH features between 1800 and 1600 cm^–1^ and concomitant appearance and growth of the –COO^–^Ag^+^ band around 1533–1536 cm^–1^ in the FTIR spectra of the respective channel recorded before and after the application of the voltage bias. Fc(exp) and Fc(calc) values obtained in this manner following 30 s and 48 h of current flow are displayed in Figure 5 alongside the corresponding FTIR spectral curves. As reported before,^21^ most spectral features representing exchangeable protons at laterally H-bonded oligomeric –COOH sites (∼1700–1600 cm^–1^) are seen to disappear in all channel setups within 30 s after the application of the voltage bias, whereas ca. 10–25% of the total carboxylic acid protons, at isolated –COOH sites represented by the residual monomeric acid band around at 1736–1732 cm^–1^ following 48 h of current flow, are not displaced with a voltage bias of 1 mV regardless of channel’s sheet resistance (*r*_s_, Figure 4), time of current flow, and total passed charge, which may exceed by far that needed for complete replacement of all carboxylic acid protons.

Considering that new silver ions entering the channel may displace, besides protons, also silver ions from –COO^–^Ag^+^ sites already generated in previous ion exchange steps, one would expect that Fc(exp) ≤ Fc(calc). Even smaller Fc(exp) values should be expected if the measured currents involve also electronic components that do not contribute to the ion exchange process. Contrary to these expectations, however, it was found that Fc(exp) ≫ Fc(calc) following 30 s of current flow (Figure 5a–c). The Fc(exp) values of the poorly conducting channels, OTSox@OTS/NTSox/Si (Figures 4b and 5b), and OTSox@OTS/NTS_OH_/Si (Figures 4c and 5c), exceed the corresponding Fc(calc) values by, respectively, 4 and 3 orders of magnitude! Likewise, Fc(exp) of the OTSox@OTS/Si channel (Figures 4a and 5a) is also almost threefold higher than the corresponding Fc(calc). Thus, the number of acid protons replaced by Ag^+^ ions following short times of current flow actually exceeds by far the total charge passed through these channels according to the measured currents and times of current flow, the discrepancy between Fc(exp) and Fc(calc) being larger the lower the measured currents (higher channel resistances). In the case of the best conducting channel setup, OTSox@OTS/PVA/Si (Figure 4d), this trend could not be presently checked, as here Fc(calc) = 1.02 (following 30 s of current flow) exceeds both the entire area of the channel and the maximal Fc(exp) value (0.90) reached after 48 h of current flow (Figure 5). As Fc(exp) ≤ 1, whereas Fc(calc) may grow indefinitely with the time of current flow, the inequality Fc(exp) < Fc(calc) must ultimately hold for sufficiently long times of current flow. Indeed, following 48 h of current flow, all Fc(exp) < Fc(calc), except for the poorest conducting setup (Figure 5b), where the total charge transported during 48 h of current flow would not replace more than 10% of channel’s protons.

The much larger Fc(exp) compared to Fc(calc) values following short times of current flow is a surprising result. It implies an ion exchange mechanism whereby the number of silver ions replacing protons exceeds by far the total charge transported through the channel according to the measured current and time of current flow. This means that a considerable portion of the mobile cations involved in the ion exchange process actually move against the direction of the electric field, thus counterbalancing the flow of charge responsible for the measured current. This further implies that fast ionic diffusion must play a major role besides the drift of ions in the direction of the electric field. Considering that no ion exchange was observed to occur in the absence of a voltage bias, whereas those 10–25% residual acid protons that may not be displaced at a bias of 1 mV (Figure 5, curves 1 mV, 48 h) are easily replaced by Ag^+^ ions at a bias of 100 mV,^21^ suggests that the generation of mobile cations itself is a process dependent on the presence of an electric field and its magnitude. Once in a free ionic state upon the application of a voltage bias, both H^+^ and Ag^+^ ions may drift as well as rapidly diffuse, thus establishing an even ionic distribution across the entire OTSox path. Further support to such a bias-dependent fast ion diffusion scenario comes from the observations that (i) virtually identical distributions of –COOH and –COO^–^Ag^+^ spectral features have always been recorded at any position of the IR beam along an OTSox path between anode and cathode, regardless of channel’s sheet resistance and time of current flow; (ii) the extent of ion exchange in the poorly conducting channels following 30 s of current flow (Figure 5b,c) significantly exceeds that in the basic OTSox@OTS/Si channel (Figure 5a) which is a far better electrical conductor.

According to these results, the ion exchange in macrochannels appears to be largely the result of fast lateral diffusion of mobile cations generated upon the application of a voltage bias rather than that of ionic charge transport according to the measured currents and times of current flow. The lack of correlation between electrical conduction and ion exchange further implies that the measured currents may actually include both ionic and electronic components; however, as confirmed by the control experiments discussed in the following, the presence and continuous supply of mobile cations along the OTSox path constitutes a necessary condition for either mode of conduction.

We finally note that the sheet resistance of OTSox@OTS/Si macrochannels (Figure 4a) is more than 3 orders of magnitude higher than that of OTSox@OTS/Si nanochannels on same silicon substrate (Figure 3a). Whether this has to do with a predominantly ionic mode of conduction in the former and electronic in the latter, possibly related to their very different dimensions and *l*/*w* aspect ratios,^21^ is an issue that has yet to be further investigated.

### Control Experiments

Effective confinement of the charge transport to the top –COOH surface of OTSox in the OTSox@OTS channels is presumably achieved owing to the good electrical insulation provided by the dense hydrocarbon core of OTSox (which blocks significant leakage currents to the underlying silicon substrate^34^) along with the virtual absence of pinholes/structural defects at the boundary between the patterned OTSox paths and the unmodified OTS monolayer^1,21^ that acts as both a perpendicular and lateral insulator. This is confirmed by a series of control experiments that provide conclusive evidence for the absence of significant leakage currents compared to those attributed to charge transport along the patterned channels.

#### Leakage Currents through the Hydrocarbon Core of the Monolayer

The insulating performance of the as-formed OTS monolayers in each of the setups investigated in this study was checked by applying a voltage bias of 1–100 mV between silver electrodes deposited on the monolayer at different distances from one another and measuring the resulting current/resistance. Typical resistance values in the range (2–50) × 10^9^ Ω obtained in these experiments correspond to residual leakage currents through the hydrocarbon core of the monolayer at least 4 orders of magnitudes smaller than those recorded from the different investigated OTSox@OTS channels (with nano-to-macrosize dimensions) on the respective substrates.

#### Broken Line Nanochannels

Conclusive evidence for the effective confinement of charge transport to continuous OTSox surface paths connecting the electrodes to which the bias is applied was obtained from a study of discontinuous nanochannels, patterned as sequences of OTSox line segments with variable lengths, separated from one another by variable insulating OTS gaps (Figure S7). Down to nominal OTS gaps of 20 nm between the OTSox segments (as defined in the IEBL design), all such broken line nanochannels were found to display resistance values of the order of (3–4) × 10^9^ Ω regardless of the distance between the electrodes, the lengths of the OTSox segments and intersegment gaps. Such resistance values are characteristic of unpatterned OTS/Si monolayers, corresponding to residual leakage currents through the hydrocarbon core of the monolayer of the order of (2–3) × 10^–13^A at 1 mV (vide supra). Broken line nanochannels with nominal intersegment gaps of 10 nm or less were found to display resistance values according to the plots in Figure 3 (currents higher than 10^–8^ A at 1 mV), i.e., like those patterned as continuous OTSox lines. Thus, as far as the electrical conduction is concerned, broken OTSox lines with nominal intersegment gaps of 10 nm or less are indistinguishable from continuous OTSox lines written under the same IEBL conditions. This sets ca. 10 nm as the practical resolution achieved in the IEBL patterning of the present OTSox/Si lines.

#### Reversible –COOH ↔ –COOR Chemical Modification of the Surface Functionality along a Portion of the Patterned OTSox Path

The ionizable –COOH surface functions of OTSox may be converted to nonionic –COOR ester moieties via the spontaneous formation of covalent ester linkages with the alcohol groups of a thin PVA film (several nanometers-thick) deposited on the OTSox surface.^1,13^ It was found that converting –COOH to –COOR in this manner on just a limited portion of the conducting path of a OTSox@OTS/Si macro- or nanochannel is sufficient to totally suppress its electrical conduction. Such partially PVA-covered channels exhibit resistance values indistinguishable from those of the insulating OTS/Si monolayer surrounding the patterned OTSox paths. Their electrical conduction is then fully restored upon the hydrolytic removal of the PVA coating, which regenerates the –COOH surface functionality.^1,13^ These experiments demonstrate the role of ionic transport as a *sine qua non* for electrical conduction in the OTSox@OTS channels, while offering further conclusive evidence for both the confinement of charge transport to the –COOH surface paths connecting the electrodes to which the bias is applied and the absence of significant leakage currents compared to those ascribed to transport along the OTSox paths.

#### Reversible –COOH ↔ –CH_2_OH Chemical Transformation of Channel’s Surface Functionality

The key role played by the ionizable –COOH surface groups of OTSox in the electrical conduction of OTSox@OTS/Si channels was finally assessed by measurements of electrical resistance performed before and after chemical reduction of the as-patterned –COOH surface functionality to the corresponding nonionic terminal alcohol (−CH_2_OH), and then following chemical oxidation of –CH_2_OH back to –COOH (Methods).

To provide unequivocal evidence for the outcome of the reduction and oxidation operations, these experiments were conducted in parallel with both macrochannels and nanochannels on the high-resistivity p-silicon. According to FTIR spectra collected from a macrochannel undergoing the chemical reduction and oxidation processes (Figure S8), all carboxylic acid features around 1700 cm^–1^ (Figure S8, curve 2) disappear following the reduction with BH_3_·THF (Figure S8, curve 3) and reappear with somewhat enhanced intensity after the back oxidation with KMnO_4_/H_2_SO_4_ (Figure S8, curve 4), while no measurable changes are observed upon any of these transformations in the intensities, widths and peak positions of the –CH_2_– stretch bands around 2017 and 2849 cm^–1^ representing the hydrocarbon tails of OTSox (Figure S8, curves 2–5). The conversion –COOH ↔ –CH_2_OH is thus quantitative and occurs with full preservation of the ordered structure of the monolayer. The ca. 1.5-fold higher integrated intensity of the –COOH features around 1700 cm^–1^ following the chemical oxidation with permanganate (Figure S8, curve 4 compared to curve 2) along with the higher proportion of laterally H-bonded oligomeric acid species (band tail extending below ∼1680 cm^–1^) suggest that (i) a fraction of the methyl groups of OTS are incompletely oxidized in the IEBL process, to alcohol rather than to carboxylic acid, so that chemical oxidation of these –CH_2_OH groups adds –COOH groups to those initially generated by IEBL; (ii) the chemical transformations –COOH → –CH_2_OH → –COOH further facilitate a certain reorganization/reorientation of the –COOH groups that favors formation of a higher proportion of laterally H-bonded carboxylic acid species. These, however, have no effect on the residual fraction of acid protons that are not replaced by Ag^+^ ions at a voltage bias of 1 mV (Figure S8, residual –COOH band around 1735 cm^–1^ in curve 5). If charge transport along a patterned OTSox path depends on the density and extent of lateral H-bonding of its –COOH groups (vide supra),^21^ these results imply that the conversion –COOH → –CH_2_OH should totally suppress electrical conduction, whereas the back conversion –CH_2_OH → –COOH should restore and enhance it compared to that measured before these chemical transformations. These expectations have been confirmed experimentally, though not without further unexpected findings as to the extent of enhancement of the electrical conduction of nanochannels following their chemical manipulation.

As expected, all tested channels completely lose their electrical conduction upon the reduction of –COOH to –CH_2_OH, the more than 6 orders of magnitude higher resistance/sheet resistance measured in the reduced state (Table 2: **Macro** 2. BH_3_ vs **Macro** 1. IEBL; **Nano** 2. BH_3_ vs **Nano** 1. IEBL) equaling that of the insulating OTS monolayer surrounding the patterned OTSox paths. The chemical oxidation of –CH_2_OH back to –COOH restores and enhances channels’ electrical conduction (Table 2: **Macro** 3. KMnO_4_; **Nano** 3. KMnO_4_), however, whereas macrochannel’s sheet resistance drops to a value ca. 2.5-fold lower than that measured before the reduction and oxidation treatments (0.51 × 10^3^ vs 1.30 × 10^3^ Ω), the ca. 26-fold drop in nanochannel’s sheet resistance (0.043 vs 1.109 Ω) is an order of magnitude larger than that of the macrochannel. To verify that this unexpectedly large enhancement of nanochannel’s conduction following the –CH_2_OH back oxidation to –COOH does not stem from a large leakage current caused by the eventual deterioration of nanochannel’s structure under the conditions of the oxidation reaction, the reduction and oxidation operations were repeated once again on same OTSox line. The essentially identical results obtained after the second reduction and oxidation operations (Table 2: **Nano** 4. BH_3_ and **Nano** 5. KMnO_4_, respectively), offer conclusive evidence for both the full preservation of nanochannel’s structure and the genuine large enhancement of its electrical conduction following the chemical oxidation operation.

**Table 2:** Resistance (*R*) and Corresponding Sheet Resistance (*r*_s_) Data Collected from a Macrochannel and a Series of Nanochannels (Derived from Same OTSox Line) after Their IEBL Patterning, after Chemical Reduction (BH_3_) of –COOH Surface Functions to –CH_2_OH, and Back Oxidation (KMnO_4_) of –CH_2_OH to −COOH

channel *l*/*w*	operation	surface function	*R* (Ω)	*r*_s_ = *R*/(*l*/*w*) (Ω)
**Macro** 3.385	1. IEBL oxidation	–COOH	4.41 × 10^3^	1.30 × 10^3^
**Macro** 3.385	2. BH_3_ reduction	–CH_2_OH	27.6 × 10^9^	8.15 × 10^9^
**Macro** 3.385	3. KMnO_4_ oxidation	–COOH	1.74 × 10^3^	0.51 × 10^3^
**Nano** 23.81 × 10^3^	1. IEBL oxidation	–COOH	26.4 × 10^3^	1.109
**Nano** 16.03 × 10^3^	2. BH_3_ reduction	–CH_2_OH	28.5 × 10^9^	1.78 × 10^6^
**Nano** 26.34 × 10^3^	3. KMnO_4_ oxidation	–COOH	1.12 × 10^3^	0.043
**Nano** 16.84 × 10^3^	4. BH_3_ reduction	–CH_2_OH	50.2 × 10^9^	2.98 × 10^6^
**Nano** 59.15 × 10^3^	5. KMnO_4_ oxidation	–COOH	2.41 × 10^3^	0.041

In view of these results, pointing to the presence of a fraction of –CH_2_OH groups (incompletely oxidized –CH_3_ groups) in the as-patterned OTSox paths, we have carried out an additional series of experiments, whereby the oxidation operation with KMnO_4_/H_2_SO_4_ was applied to as-patterned channels, skipping the reduction with BH_3_·THF. The effects observed in these experiments are similar though somewhat weaker, with a 1.2–1.3-fold growth of the integrated intensity of the –COOH features around 1700 cm^–1^ following the chemical oxidation and a corresponding ca. 1.35-fold drop in the sheet resistance of the respective macrochannel. The ca. 20-fold drop in a nanochannel’s sheet resistance following its chemical oxidation is again more than an order of magnitude larger than that of the macrochannel exposed to the same chemical treatment, though somewhat smaller than that of the nanochannel undergoing both the reduction and oxidation operations (vide supra).

Vis-à-vis what appears as relatively modest changes (as revealed by FTIR spectroscopy) in the density and organization of macrochannels’ –COOH groups upon their postpatterning chemical manipulation by reduction and oxidation or by only oxidation, the very large enhancements of nanochannels’ electrical conduction following these chemical modifications is another unexpected finding that remains to be further investigated.

## Conclusions

Surface channels with –COOH functionality fabricated by the IEBL patterning of insulating OTS/Si monolayers are artificial single-layer structures that exhibit unusual electrical conduction upon the application of a small lateral voltage bias to pairs of silver electrodes deposited at variable distances from one another along the patterned channel path. The combined analysis of electrical, AFM and FTIR spectral data obtained with such surface channels points to a complex mechanism of electrical conduction in these single-layer entities, apparently involving coupled ionic-electronic transport mediated and modulated by interfacial interactions with charge carriers located outside the conducting channel and separated from those carrying the measured current.

Attributing nanochannels’ conduction mainly to electronic transport within the ca. 1 nm-thick silver metal layer formed by the electrochemical reduction of mobile Ag^+^ ions released by the electrodes upon the application of a voltage bias, which replace –COO^–^H^+^ protons along the patterned OTSox paths, the room-temperature conduction of such metalized nanochannels on a high- and a low-resistivity p-silicon substrate was found to exceed that of the bulk silver metal by factors of, respectively, 7–43 and 24–124. The range of variation of these figures corresponds to apparent variations of the cross sections of the respective metalized nanochannels as estimated from their AFM images. However, our analysis strongly suggests actual confinement of the electronic transport to an ultrathin conduction path possibly not thicker than one-to-few atomic layers of silver, which would place the conduction enhancement close to the high rather than the low limit of each estimated range. A further up to 26-fold enhancement of the electrical conduction of nanochannels on the high-resistivity silicon substrate was achieved upon postpatterning chemical reduction of their –COOH surface functions to –CH_2_OH and back oxidation to –COOH, thus reaching a more than 3 orders of magnitude enhancement of their electrical conduction compared to that of the bulk silver metal.

We note two salient features of this phenomenon of enhanced electrical conduction: (i) it occurs in ultrathin nanowire-like metal entities on p-silicon substrates separated from the silicon surface by a 3.6–4.0 nm-thick dielectric barrier (composed of the 2.3 nm-thick hydrocarbon core +0.3 nm-thick silane headgroup of the organic monolayer + the 1.0–1.4 nm-thick native silicon oxide^32,35^); (ii) the conduction enhancement is higher the lower the resistivity of the silicon substrate, i.e., the higher the concentration of hole carriers in the substrate. These findings point to a conduction mechanism that may not be rationalized within the framework of conventional charge transport mechanisms, possibly involving interfacial electrical interactions akin to those invoked in the proposed mechanisms of excitonic superconductivity,^36,37^ in particular that by the Coulomb pairing of electrons and holes moving in closely spaced layers separated by a thin dielectric barrier that prevents significant interlayer tunneling.^23,38^ Theoretical and experimental efforts devoted to such and related Coulomb drag interactions that affect charge transport^39^ have considered cases of coupled electron–hole^22,38,40−42^ and electron–electron^42,43^ transport between two 2D layers, coupled transport between a 2D and a 3D electron-gas,^44^ and Coulomb coupling between two parallel 1D electron systems.^45,46^ Our conductive channels on the outer surface of an insulating organic monolayer on silicon are considerably more complex structures. These surface channels are not made of a specific material, rather being multicomponent nanosystems with modifiable composition and structure. The release and reduction of Ag^+^ ions to elemental silver upon the application of a voltage bias is a dynamic electrochemical process that induces charge transport which is in turn affected by chemical and structural modifications of the conduction path resulting from it. Intralayer and interlayer Coulomb interactions affecting the overall ionic-electronic transport involve here different mobile and immobile charged species, within the conducting channel as well as in an adjacent medium with dissimilar structure, composition, and dimensionality: mobile Ag^+^ and H^+^ ions and electrons, and immobile –COO^–^ anions confined to quasi-1D (nanochannel) or quasi-2D (macrochannel) conductive paths on the surface of the organic monolayer and mobile/immobile charges (holes, electrons) in the 3D silicon substrate. While theoretical models that may deal with the structural-chemical complexity of such composite systems are yet to be advanced, our findings offer experimental support to the possible role played in the observed enhancement of the electronic conduction by the Coulomb coupling of electrons confined to the patterned surface paths and holes in the silicon substrate, the two being separated by a thin dielectric barrier that prevents significant interfacial charge passage.^23,38^ These observations guide ongoing efforts toward realization of related interfacial systems that would both reach ever lower resistivities and provide further clues to their mechanisms of enhanced electrical conduction.

## Methods

High quality OTS/Si monolayers were prepare as described before^1,13,47^ on double-side polished silicon wafer substrates (0.5 mm thick, p-type, ⟨100⟩, resistivity 8–11 or 1–5 × 10^–3^ Ω cm) covered with their native oxide layer. OTS/NTSox/Si and OTS/NTS_OH_/Si bilayers were prepared as described in ref (32). The postpatterning chemical reduction of the –COOH functions of OTSox to –CH_2_OH with BH_3_·THF and the back oxidation of –CH_2_OH to –COOH with KMnO_4_/H_2_SO_4_ were performed following the procedures detailed in ref (32).

The IEBL patterning with a thin PVA film as solid oxidant was performed using the methodology described in refs (1) (nanoscale) and (13) (macroscale). Millimeter-long OTSox lines with uniform minimal width were patterned using the FBMS mode of the Raith e-beam writer, whereby the stage holding the to-be-patterned OTS/Si specimen is moved relative to the electron beam. With a line width input of 0 nm, this patterning mode may yield the narrowest OTSox lines for a given beam energy and beam current (determined by aperture and beam energy) by proper adjustment of the stage speed, which sets the exposure line dose. Typical optimal IEBL conditions (affording nondestructive quantitative conversion of –CH_3_ to –COOH) employed in the FBMS writing of such OTSox lines: PVA/OTS film coating ca. 5 nm-thick (deposited from 0.1% aqueous solution^1,13^), beam energy 20 keV, aperture 7 μm, stage speed 0.2 mm/s.

AFM semicontact (tapping) and contact mode images were acquired as described before,^1^ on an NTEGRA Aura System (NT-MDT) purged with dry nitrogen withdrawn from liquid N_2_. The half-height line widths obtained from AFM cross-section profiles were taken as reasonable estimations of the respective nanochannel widths.

Quantitative Brewster’s angle FTIR spectra^24^ (4 cm^–1^ resolution) were acquired as described before^13,21^ on a Bruker Equinox 55 spectrometer equipped with a liquid nitrogen-cooled MCT detector, a KRS-5 wire grid polarizer and a computer-controlled shuttle accessory, purged with dry nitrogen withdrawn from liquid N2. All displayed spectral curves represent net spectra of the respective monolayers on the front side of the double-side polished silicon wafer substate, after mathematical subtraction of spectral contributions of the silicon substrate and of the unmodified OTS monolayer on the back side of the substrate.

Electrical transport measurements were performed with a Solartron Modulab system (Ametek, Solartron Analytical) using a special homemade probe station equipped with PogoPlus snap-out spring probes with beryllium–copper alloy, rhodium plated over hard nickel tips, purged with pure nitrogen withdrawn from liquid N_2_ (RH ≈ 2%).^21^ Resistance data were acquired at the ambient temperature (21 ± 0.5 °C) by recording the current through the channel upon the application of a dc voltage bias of ∼1.0 mV to the silver electrodes deposited on the respective OTSox line or rectangle (Figure 1).

Silver electrodes (ca. 50 nm-thick) were prepared by thermal metal deposition through contact masks under carefully controlled conditions that do not damage the organic monolayer. To avoid leakage currents possibly caused by penetration of probe tips into the electrodes, all electrodes were equipped with soft Ag/PVA contacts (Figure 1) prepared by deposition of the metal onto thick PVA pads prepared by pulling liquid with a fine needle from a droplet of a viscous PVA solution in water and letting then the water evaporate in a dust-free atmosphere. The same procedure was used to cover portions of OTSox lines with protective PVA stripes that define the lengths of the respective nanochannels during the deposition of the electrodes. Subsequent peeling off the PVA stripe lives a clean –COOH nanochannel surface, free of contamination, and silver particles that may spread under the masks during metal evaporation.

## Supporting Information Available

Supplementary AFM images, supplementary explanatory schemes, and supplementary FTIR spectra (PDF)
