# Supplementary material for: Interfacial Electron Beam Lithography Converts an Insulating Organic Monolayer to a Patterned Single-Layer Conductor with Puzzling Charge Transport Performance
Source: ACS Nano. 2024 Jul 9;18(29):18948–62. doi: 10.1021/acsnano.4c02074 (PMC11271180; doi:10.1021/acsnano.4c02074)
Supplement: Supplementary file 1 — nn4c02074_si_001.pdf [file nn4c02074_si_001.pdf]

Supporting Information

Interfacial Electron Beam Lithography Converts an Insulating Organic Monolayer to a Patterned Single-Layer Conductor with Puzzling Charge Transport Performance

Rivka Maoz,* Peter Nelson,† Bedanta Gogoi,† Doron Burshtain, Santanu Talukder, Shuangyang Zou, Arup Sarkar, Jonathan Berson, and Jacob Sagiv*

Department of Molecular Chemistry and Materials Science, Weizmann Institute of Science, Rehovot 7610001, Israel

*Email: rivka.maoz@weizmann.ac.il jacob.sagiv@weizmann.ac.il

†Equally contributing authors

Present Address:

P.N. *Department of Chemistry, Faculty of Science and Technology, The University of the West Indies Mona, Kingston 7, St. Andrew, Jamaica*

B.G. *Department of Chemical Sciences, Tezpur University, Assam 784028, India*

S.T. *Department of Electrical Engineering and Computer Science, Indian Institute of Science Education and Research, Bhopal Bypass Road, Bhopal, Madhya Pradesh 462066, India*

S.Z. *Key Laboratory of Microgravity, Institute of Mechanics, Chinese Academy of Sciences, Beijing 100190, China*

J.B. *Institute of Applied Physics, Karlsruhe Institute of Technology, Wolfgang-Gaede-Strase 1, 76131 Karlsruhe, Germany; Institute of Nanotechnology, Karlsruhe Institute of Technology, Hermann-von-Helmholtz-Platz 1, 76344 Eggenstein-Leopoldshafen, Germany*

Supplementary AFM images

Figure S1, **Figure S2**, **Figure S3**, **Figure S7**

Supplementary explanatory schemes

Figure S4, **Figure S5**

Supplementary FTIR spectra

Figure S6, **Figure S8**

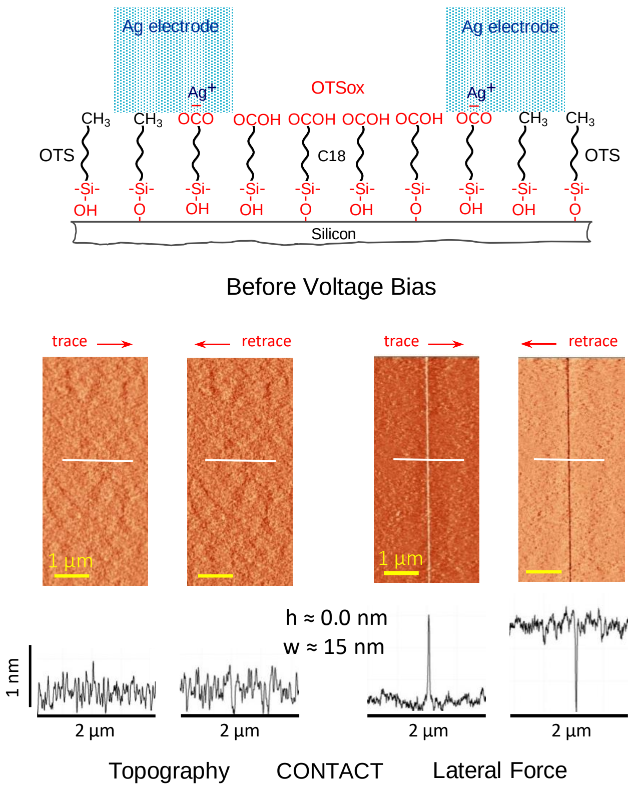

Figure S1. Contact mode topography and lateral force AFM images of the OTSox@OTS/Si nanochannel in Figure 2a of the main text (written with a line width input of 0 nm on the high-resistivity Si substrate) showing both the trace and retrace scans (only the trace scan images are displayed in Figure 2a).

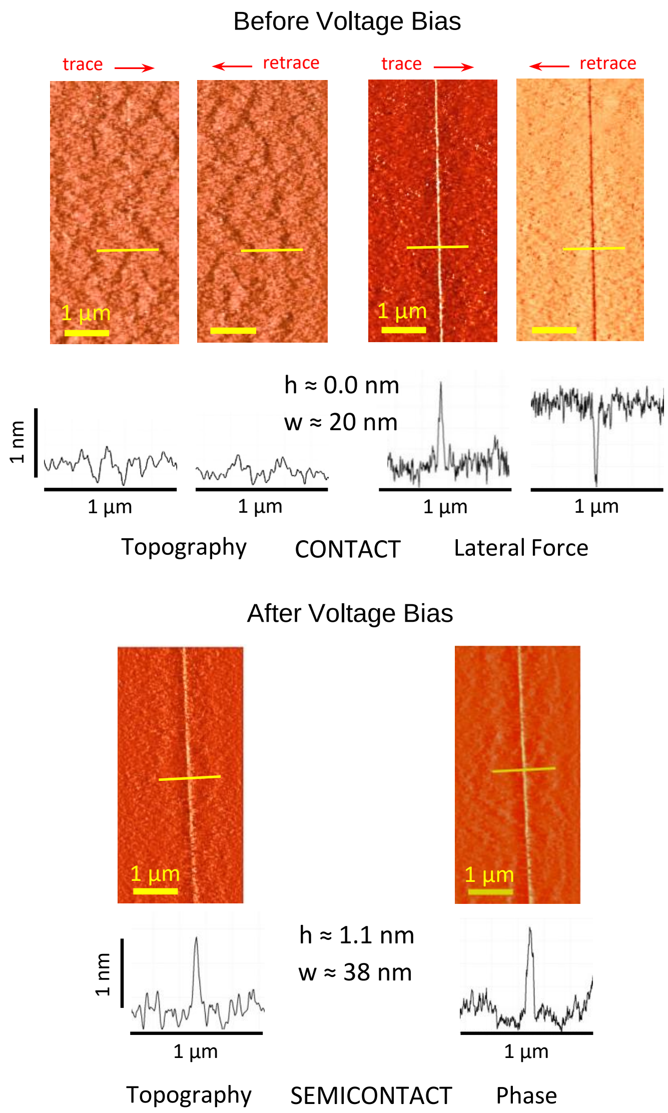

Figure S2. Complete set of contact and semicontact AFM images and cross-section profiles of portions of a OTSox@OTS/Si nanochannel written with a line width input of 0 nm on the low-resistivity p-Si substrate. The contact mode images (top) were obtained before current flow through the channel and the semicontact mode images (bottom) after current flow.

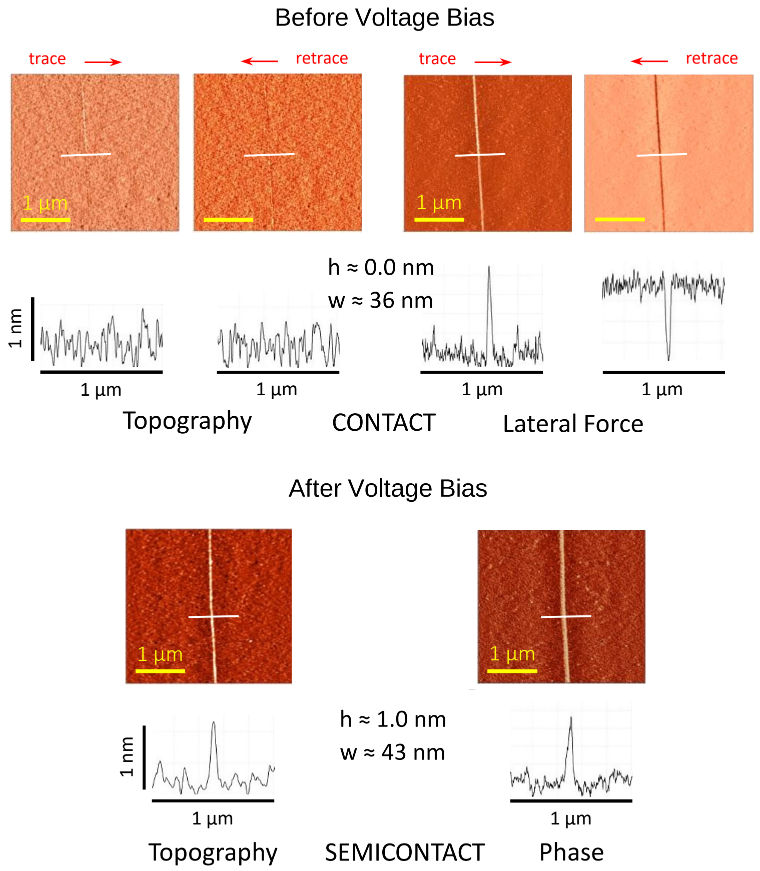

Figure S3. AFM images as in Figure S2 of a OTSox@OTS/Si nanochannel written with a line width input of 50 nm on the high-resistivity p-Si substrate.

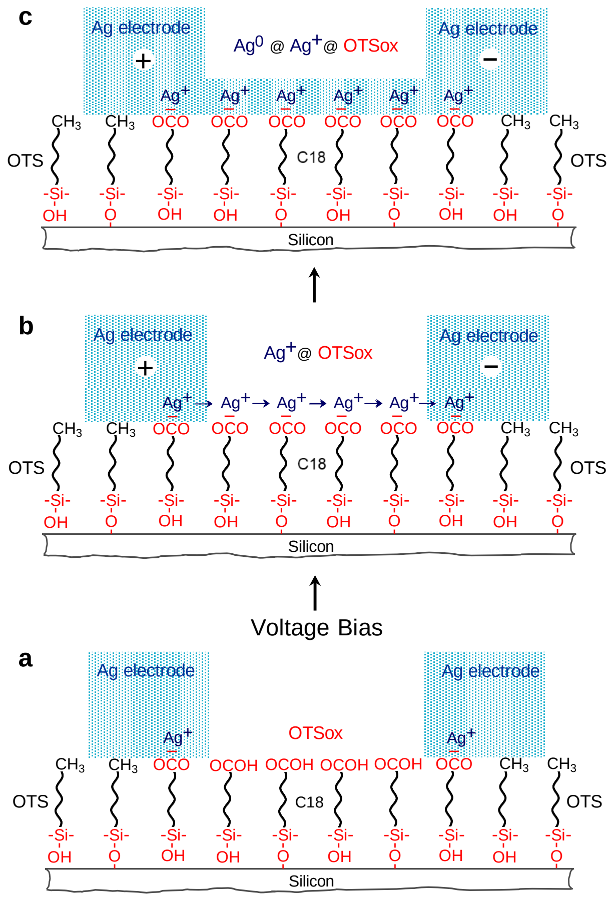

Figure S4. Schematic molecular side-views of: (**a**) OTSox@OTS/Si nanochannel with silver electrodes before the application of a voltage bias (OTSox: -COOH surface), (**b**) mobile silver ions replacing carboxylic acid protons upon the application of a voltage bias between the silver electrodes ($-COO^{-}Ag^{+}$ surface: Ag^{+}@OTSox), (**c**) nanowire-like silver metal deposit connecting the silver electrodes, formed upon the reduction of Ag^{+} ions moving on the $-COO^{-}$ surface (Ag^{0} on $-COO^{-}Ag^{+}$ surface: Ag^{0}@Ag^{+}@OTSox). Note that the preservation of electroneutrality demands presence of unreduced Ag^{+} ions within the deposited silver metal in amount equal to that of the immobile $-COO^{-}$ surface functions.

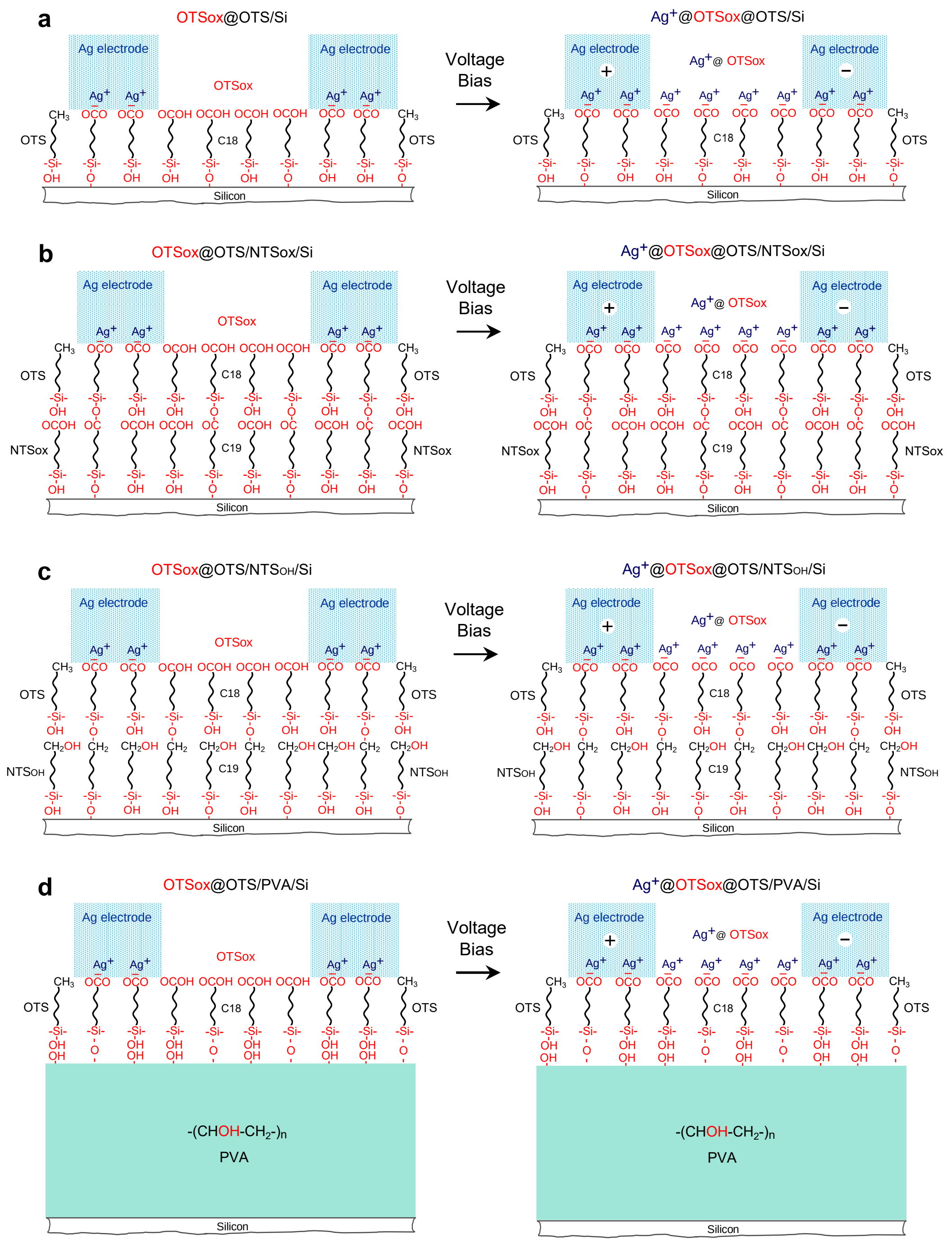

Figure S5. Schematic of the replacement of carboxylic acid protons by silver ions upon the application of a voltage bias between the silver electrodes in conducting OTSox@OTS/ISL/Si macrochannel setups with different ISLs, as in Figure 4 of the main text: (**a**) OTSox@OTS/Si: ISL-free macrochannel, (**b**) OTSox@OTS/NTSox/Si: bilayer setup with NTSox monolayer as

ISL, (**c**) OTSox@OTS/NTSOH/Si: bilayer setup with NTSOH monolayer as ISL, (**d**) OTSox@OTS/PVA/Si: bilayer setup with a thin PVA film as ISL.

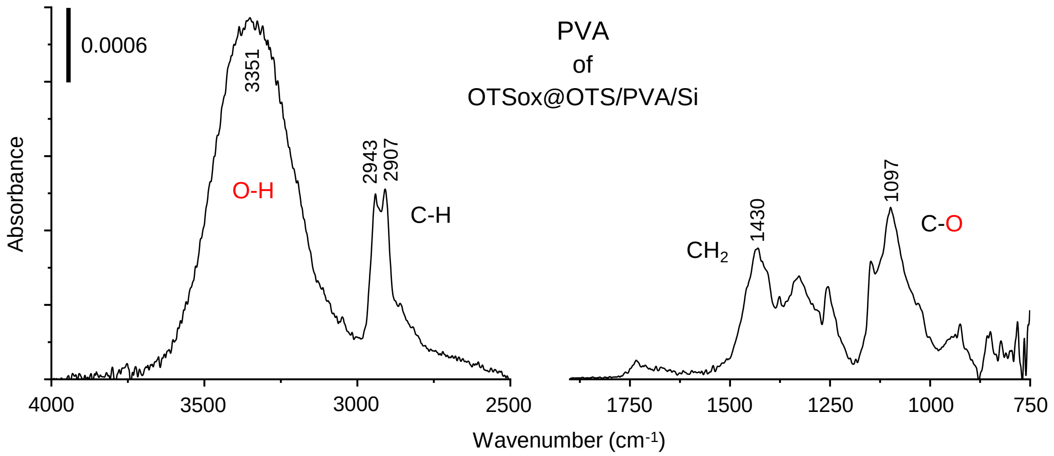

Figure S6. Brewster's angle FTIR spectrum of the PVA film used as ISL in the OTSox@OTS/PVA/Si macrochannel setup in Figure S5 and Figures 4 and 5 of the main text. As in Figure 5 of the main text, this is a difference spectral curve representing the net spectral contribution of the PVA film, after mathematical subtraction of the contributions of the silicon substrate to the measured PVA/Si spectrum.

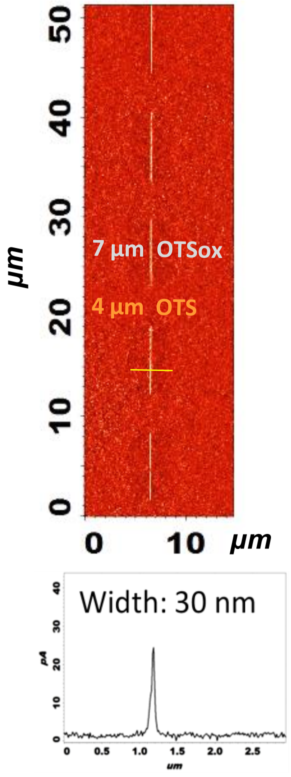

Figure S7. Example of lateral force AFM image of a portion of a broken line nanochannel with 7 μm-long conducting OTSox segments separated from one another by 4 μm-long insulating OTS gaps.

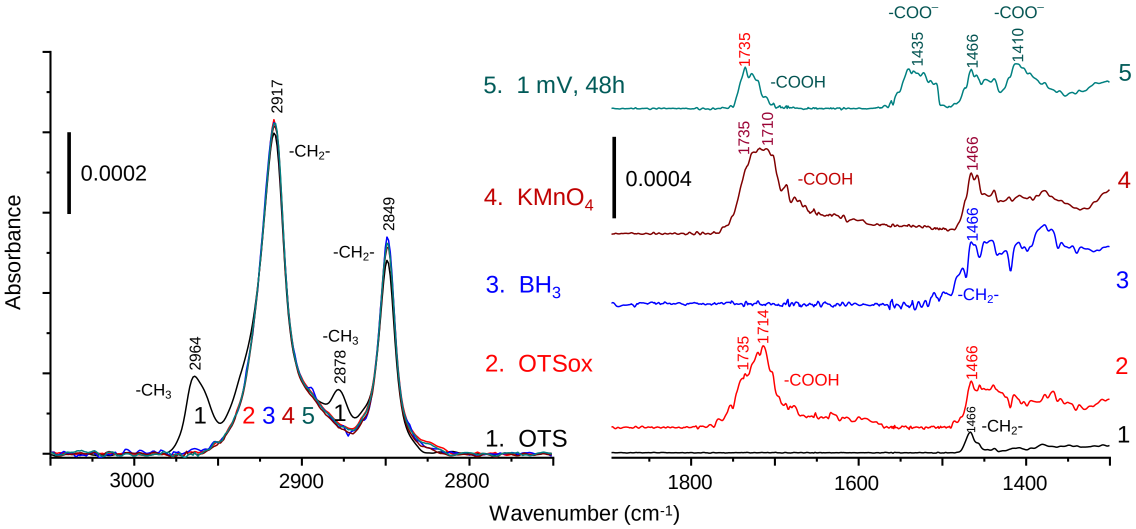

Figure S8. Quantitative Brewster's angle FTIR spectra (as in Figure 5a of the main text) collected from a OTSox@OTS/Si macrochannel at consecutive stages of its patterning and post-patterning chemical manipulation by reduction with BH_3·THF, then oxidation with $KMnO_4/H_2SO_4$, and following current flow through the channel after the oxidation step. 1. OTS: monolayer before patterning; 2. OTSox: patterned OTSox channel region; 3. BH_3: OTSox channel region after chemical reduction; 4. $KMnO_4$: OTSox channel region after chemical reduction followed by oxidation; 5. 1mV, 48h: OTSox channel region after the reduction and oxidation operations followed by current flow for 48 hours at a voltage bias of 1 mV (see text).
